# Dietary and metabolic reprogramming alleviates neurodegeneration: a review of mechanisms and clinical implications

**DOI:** 10.3389/fnut.2026.1706597

**Published:** 2026-01-29

**Authors:** Liyuan Fang, Yafei Zhuang, Minli Zhang, Deqian Yang, Ruyi Zhang, Jusheng Peng, Changhua Wang

**Affiliations:** 1 Wuhan Institute of Design and Sciences, Wuhan, China; 2 School of Pharmacy, Xianning Medical College, Hubei University of Science and Technology, Xianning, Hubei, China; 3 School of Basic Medical Sciences, Xianning Medical College, Hubei University of Science and Technology, Xianning, China; 4 School of Pharmacy, Hubei University of Chinese Medicine, Wuhan, China; 5 Department of Obstetrics and Gynecology, Xianning First People's Hospital of Hubei Province, Xianning, China

**Keywords:** cellular metabolic reprogramming, mitochondrial dysfunction, neurodegenerative disease, nutritional intervention, personalized nutrition

## Abstract

Neurodegenerative diseases, characterized by their insidious onset and progressive neuronal degeneration, present significant challenges in the fields of neuroscience and medicine. We elucidate the critical role of nutrition and cellular metabolism in the pathogenesis and progression of these disorders, with a particular focus on Alzheimer’s disease (AD), Parkinson’s disease (PD), and Huntington’s disease (HD). We demonstrate that fundamental nutrients such as glucose, lipids, and amino acids are crucial for neuronal bioenergetics, oxidative stress mitigation, and neuroprotective functions. Furthermore, we emphasize the concept of metabolic reprogramming as a key driver in neurodegeneration; this process entails alterations in energy metabolism, mitochondrial dysfunctions, and shifts in nutrient utilization that exacerbate neuroinflammation and oxidative stress. We emphasize the potential advantages of nutritional strategies, especially those involving the Mediterranean dietary pattern, characterized by high antioxidant and omega-3 fatty acid content, to optimize cellular metabolic pathways and attenuate disease manifestations. However, clinical application of nutritional strategies faces several challenges including complexities surrounding nutrient mechanisms, patient adherence issues, and concerns regarding long-term efficacy. To address these obstacles, we advocate for personalized nutrition approaches that integrate metabolomics, genomics, and epigenetics to tailor interventions according to individual metabolic profiles. Additionally, emerging strategies such as probiotics along with synergistic combinations of nutrients and pharmaceuticals offer promising avenues for enhancing therapeutic outcomes. In conclusion, understanding the intricate interplay between nutrition and cellular metabolism is crucial for developing effective treatments for neurodegenerative diseases. Future research should prioritize mechanistic studies alongside precise assessment tools as well as high-quality clinical trials to validate the efficacy of these interventions.

## 1 Introduction

Neurodegenerative disorders, including Alzheimer’s disease (AD), Parkinson’s disease (PD), and Huntington’s disease (HD), are characterized by progressive neuronal dysfunction and cell death, leading to irreversible cognitive and motor decline (1–3). These conditions not only severely impair quality of life but also place a growing burden on global healthcare systems (4). Despite substantial advances in understanding their pathophysiology, effective disease-modifying therapies remain limited, highlighting the urgent need for novel therapeutic strategies.

Emerging evidence underscores the central role of nutrient metabolism in maintaining neuronal integrity. Neurons rely heavily on glucose and lipid metabolism to meet high energy demands, while amino acid metabolism supports neurotransmitter synthesis and synaptic plasticity (5–7). In neurodegenerative diseases, these metabolic processes are frequently disrupted. For instance, impaired glucose utilization and insulin resistance are hallmarks of AD, contributing to energy deficits and oxidative stress (8–11). Similarly, mitochondrial dysfunction and lipid peroxidation are key pathogenic features in PD and HD (12–17).

In this context, metabolic reprogramming, the adaptive rewiring of cellular metabolic pathways in response to stress or disease, has gained recognition as a critical driver of neurodegeneration (18, 19). Metabolic reprogramming refers to the cellular adaptation of metabolic pathways in response to specific pathological conditions to satisfy energy requirements or to cope with environmental stressors (20, 21). These alterations not only influence the energy status of neurons but are also intricately associated with critical pathological mechanisms such as neuroinflammation and oxidative stress.

Nutritional intervention is emerging as a promising therapeutic strategy, garnering increasing attention. The Mediterranean diet, abundant in antioxidants, omega-3 polyunsaturated fatty acids, and fiber, has demonstrated efficacy in enhancing cognitive function and alleviating symptoms in individuals with neurodegenerative diseases (22). Nevertheless, the clinical application of nutritional interventions faces numerous challenges, including the complexity of nutrient action mechanisms, patient adherence, and the long-term efficacy and safety of such interventions (23). To address these challenges, future research must delve deeper into the mechanisms underlying nutritional interventions and develop more precise assessment tools and personalized treatment approaches. The integrated application of metabolomics, genomics, and epigenetics will provide a theoretical foundation for tailored nutritional interventions. Furthermore, the incorporation of probiotics and epigenetic strategies, along with combined nutrient and pharmacological treatments, has opened new avenues for the management of neurodegenerative diseases (24).

This review systematically searched the PubMed, Web of Science, and CNKI databases. The search keywords included “neurodegenerative disorders,” “metabolic reprogramming,” “nutritional therapy,” “Mediterranean diet,” “mitochondrial dysfunction,” “Alzheimer’s disease,” “Parkinson’s disease,” and “Huntington’s disease,” among others. The objective is to investigate the role of nutrition and cellular metabolic pathways in the pathogenesis of neurodegenerative conditions, elucidate their molecular mechanisms, and propose novel therapeutic interventions. By synthesizing findings across multiple scientific disciplines, the goal is to enable targeted treatment strategies for neurodegenerative diseases and to develop effective therapeutic modalities. Achieving this necessitates not only advances in basic research but also multidisciplinary collaboration among clinical medicine, nutritional science, molecular biology, and related fields to foster ongoing progress in disease management.

## 2 Nutrition and neuronal metabolism

### 2.1 Effects of nutrients on neuronal metabolism

**Figure 1:**
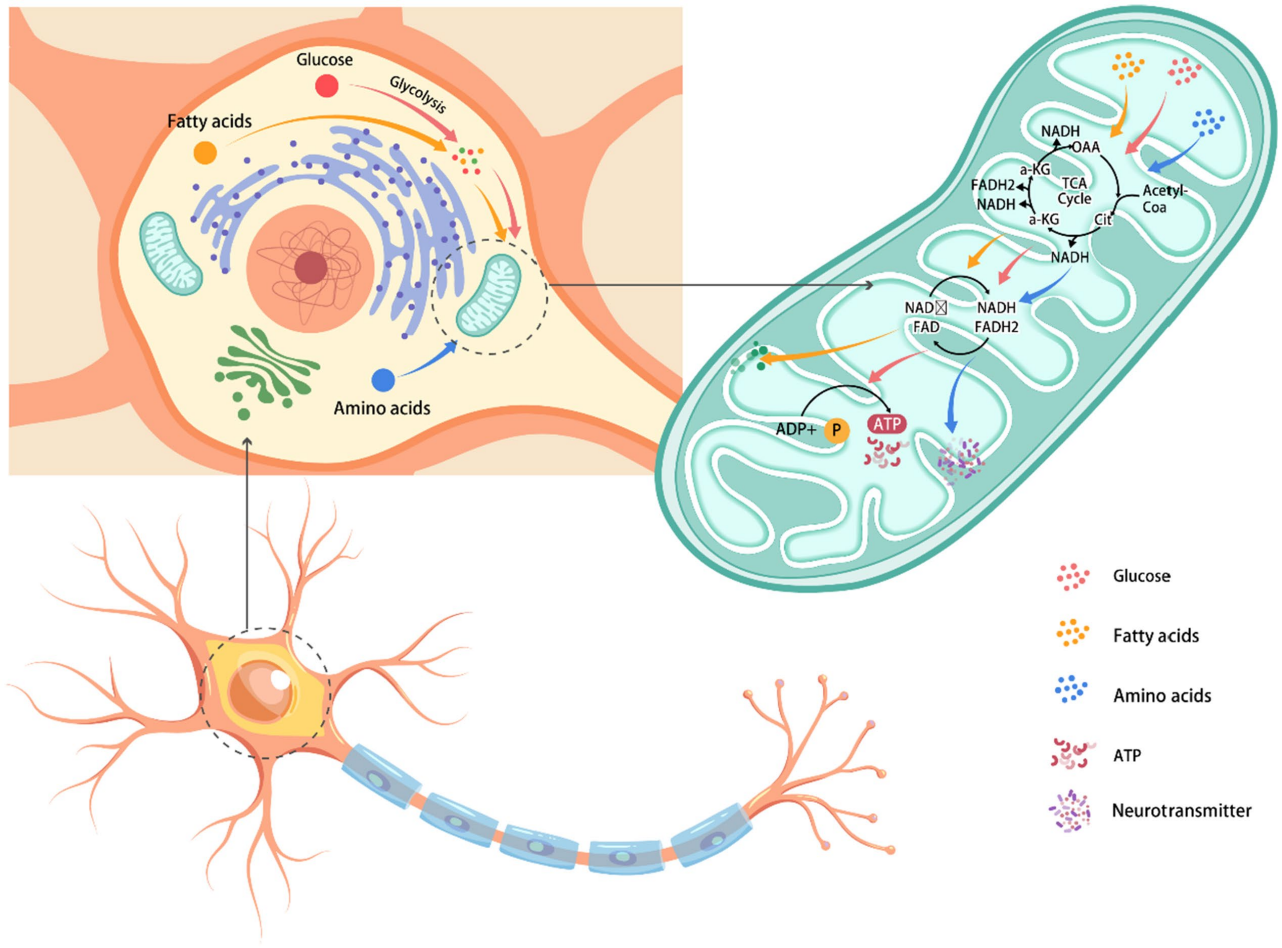
The influence of nutrients on neuronal metabolism. Glucose, fat, and amino acids in the extracellular environment enter neurons through the cell membrane. Glucose is converted into pyruvic acid through glycolysis. After entering the mitochondria, pyruvate participates in the tricarboxylic acid cycle, generating NADH and FADH_2_. These substances transfer electrons to oxygen through the electron transport chain, eventually producing ATP, which provides the main energy for the cell and generates key metabolites such as acetyl coenzyme A and citric acid. Neurons transmit signals through axons and release neurotransmitters powered by ATP at synapses.

The pivotal role of nutrients in neuronal metabolism is undeniable; they not only supply energy but also contribute to the maintenance and repair of neuronal functions (25, 26). Figure 1 summarizes nutrient effects on neuronal metabolism. Through this pathway, Neurons oxidize to 5.8 kg glucose per day (27). Through glycolytic pathways, glucose undergoes catabolism to form pyruvate, which then translocates into mitochondria to participate in the tricarboxylic acid cycle, culminating in ATP synthesis through oxidative phosphorylation; this metabolic process is essential for supplying the energy required for neuronal function (28). However, in the context of neurodegenerative diseases, disruptions in glucose metabolic pathways can hinder energy supply to neurons, resulting in dysfunction and cellular damage (29).

Lipids, particularly omega-3 polyunsaturated fatty acids like docosahexaenoic acid (DHA), are vital for maintaining the fluidity of neuronal membranes and enhancing the efficacy of nerve signal transmission (30). A deficiency in DHA has been linked to increased neuroinflammation and the progression of neurodegenerative conditions, while DHA supplementation has been shown to bolster the stability of neuronal membranes and promote neuroprotection (31–33). Furthermore, disturbances in lipid metabolism, such as impaired fatty acid oxidation, may exacerbate neurodegenerative disease progression; thus, regulating lipid metabolism could potentially alleviate neuronal damage (34).

Amino acid metabolism is also integral to neuronal function. Amino acids serve not only as the fundamental components of proteins but also as precursors for neurotransmitter synthesis (35, 36). For instance, tryptophan and tyrosine are converted into neurotransmitters like serotonin and dopamine in the brain, which are crucial for mood regulation and motor control (37–39). In neurodegenerative diseases, imbalances in amino acid metabolism can result in abnormal neurotransmitter levels, adversely affecting inter-neuronal signaling.

Within the nutrient metabolism pathways, glycolysis, the tricarboxylic acid cycle, and oxidative phosphorylation are essential processes for neuronal energy production (40, 41). Glycolysis occurs in the cytoplasm, converting glucose into pyruvate and yielding a modest amount of energy rapidly (42). The tricarboxylic acid cycle, located in the mitochondria, further metabolizes pyruvate, and integrates with oxidative phosphorylation to produce substantial ATP quantities (43). Oxidative phosphorylation, the final stage of neuronal energy metabolism, transpires in the inner mitochondrial membrane, generating ATP through electron transport chains and proton gradients (44). Disruptions in these metabolic pathways, particularly in neurodegenerative diseases, can lead to insufficient energy supply, neuronal dysfunction, and accelerated neurodegeneration.

Nutrients not only furnish energy for neurons but also play a role in preserving neuronal structure and facilitating signal transmission (45). For example, vitamins C and E function as antioxidants, shielding neurons from oxidative damage by neutralizing free radicals (46, 47). The B vitamin complex, especially folate, vitamin B6, and vitamin B12, is crucial for neurotransmitter synthesis and the formation of the myelin sheath, thereby supporting neuronal function (48, 49). Consequently, adequate nutrient intake and metabolic equilibrium are vital for sustaining neuronal health. We present a synthesis of the impact of nutrients on neuronal metabolism in Figure 1.

### 2.2 Nutrient metabolism and neuronal function

The interplay between nutrient metabolism and neuronal function represents a multifaceted biological mechanism wherein nutrients serve not only as energy sources but also as direct modulators of nerve signaling and synaptic plasticity (50). The equilibrium of metabolic pathways for nutrients significantly influences the energy status of neurons, thereby impacting their normal functionality and the stability of neural networks (51).

Nerve signaling relies on the release and reception of neurotransmitters, a process that is energetically demanding (52). Nutrients, particularly amino acids, are critical precursors in the synthesis of neurotransmitters (53). For example, the amino acids tryptophan and tyrosine are essential precursors in the biosynthesis of serotonin and dopamine, respectively; these neurotransmitters play a crucial role in modulating affective states, cognitive processes, and motor control (54–56). Disruptions in amino acid metabolism, whether through deficiency or excess, can lead to imbalances in neurotransmitter levels, impairing the normal transmission of nerve signals and potentially contributing to the pathogenesis of neurodegenerative disorders (57, 58).

The B vitamin complex, encompassing folic acid, vitamin B6, and vitamin B12, is vital for neurotransmitter synthesis and the formation of the myelin sheath (59, 60). Deficiencies in these vitamins can result in neuronal signaling disruptions, hindering inter-neuronal communication, and prolonged deficiencies may exacerbate neurodegenerative processes.

Synaptic plasticity, defined as the modulation of synaptic strength between neurons, is essential for learning and memory. Nutrient metabolism plays a crucial role in sustaining synaptic plasticity (61). For example, omega-3 polyunsaturated fatty acids, particularly DHA, directly influence the fluidity of neuronal membranes, thereby affecting the localization of synaptic proteins and the fusion of synaptic vesicles, which in turn impacts synaptic plasticity (62, 63). A deficiency in DHA has been associated with synaptic dysfunction, while DHA supplementation has demonstrated potential in enhancing learning and memory, offering avenues for early intervention and prevention of neurodegenerative diseases (64).

Neurons exhibit high energy demands, with nutrient metabolism serving as the primary energy source (65). Glucose and ketone bodies are the principal energy substrates for the brain, generating ATP through glycolysis, the tricarboxylic acid cycle, and oxidative phosphorylation (66). In neurodegenerative conditions, such as AD, there is a marked decline in cerebral glucose metabolism, leading to insufficient energy supply, neuronal dysfunction, and heightened cellular damage (67). Nutritional strategies, such as the Mediterranean diet, rich in antioxidants and omega-3 fatty acids, have the potential to enhance neuronal metabolism and augment energy supply, thereby decelerating disease progression (68).

The role of mitochondrial quality control (MQC) in maintaining neuronal health is increasingly recognized as a critical factor in the prevention and management of neurodegenerative diseases. Recent research has highlighted the intricate mechanisms by which MQC is regulated and its implications for neuronal function. Notably, the modulation of mitochondrial motility and the expression of specific proteins such as Syntaphilin and Mitofusin 2 have been shown to play pivotal roles in coordinating MQC dynamics, thereby promoting neuronal health (69). These proteins act as mitochondrial motility modulators, facilitating the appropriate quality control pathways in response to mitochondrial damage, which is crucial for neuronal survival and function.

In addition to the structural and functional maintenance of mitochondria, the role of exercise in enhancing MQC has been explored, particularly in the context of cerebral ischemia. Exercise training has been demonstrated to upregulate the expression of sirtuin-3 (SIRT3), a mitochondrial protein that is essential for maintaining mitochondrial integrity and function. This upregulation is associated with improved neural function, reduced neuronal apoptosis, and re-established MQC following ischemic events (70). The study underscores the potential of exercise as a non-pharmacological intervention to optimize mitochondrial function and suggests that SIRT3 could be targeted for therapeutic strategies aimed at mitigating brain ischemia.

### 2.3 Abnormal nutrient metabolism and neuronal injury

Abnormal nutrient metabolism, particularly in metabolic disorders like diabetes and obesity, significantly influences neuronal function and accelerates neurodegenerative processes (71–73). These metabolic conditions not only disrupt the normal metabolism of glucose and lipids but also initiate a cascade of events that result in neuronal damage and dysfunction. The interplay between metabolic stress and neuronal injury primarily involves oxidative stress, inflammatory responses, and mitochondrial dysfunction, all of which collectively exacerbate the degeneration of neurons.

There exists a robust correlation between diabetes, especially type 2 diabetes, and neurodegenerative diseases such as AD (74). In individuals with diabetes, the hyperglycemic state induces irregular energy metabolism in neurons, while the accumulation of advanced glycation end products (AGEs) intensifies oxidative stress and inflammatory responses, thereby impairing neuronal structure and function (75). Prolonged hyperglycemia can also lead to vascular complications, diminishing blood flow to neurons and further exacerbating neuronal injury (76). In diabetic patients, insulin resistance and *β*-cell dysfunction result in irregularities in insulin signaling, adversely affecting glucose utilization and energy production in neurons (77). Insulin plays a crucial role in regulating neuronal function, influencing neuronal growth, differentiation, and synaptic plasticity. Insulin resistance not only diminishes glucose uptake and utilization by neurons but also inhibits critical insulin-mediated signaling pathways such as PI3K/Akt and mTOR, which are essential for neuronal survival and functionality (77).

Obesity, particularly abdominal obesity, is linked to chronic low-grade inflammation and a state of metabolic stress that can potentially harm neurons (78). In obese individuals, various inflammatory mediators produced by adipose tissue, such as TNF-*α* and IL-6, in-filtrate the brain via the bloodstream, triggering neuroinflammatory responses that damage neurons and glial cells, thereby disrupting normal neuronal function. Furthermore, obesity is associated with lipid metabolism abnormalities, including impaired fatty acid oxidation and lipid accumulation, which exacerbate oxidative stress in neurons and promote neurodegenerative processes (79).

Mitochondrial dysfunction represents a critical mechanism underlying neuronal injury in the context of metabolic stress. Mitochondria serve as the hub of cellular energy metabolism, and their impairment leads to reduced efficiency in oxidative phosphorylation and excessive generation of reactive oxygen species (ROS) (80, 81). The elevation of ROS not only inflicts damage on mitochondrial DNA but also disrupts proteins and lipids, instigating oxidative stress that compromise’s neuronal structure and function. Additionally, mitochondrial damage activates the neuronal apoptotic pathway, further hastening neurodegeneration (82). Metabolic disorders also incite neuroinflammation, with the activation of microglia and astrocytes releasing a plethora of inflammatory mediators, such as IL-1β, TNF-*α*, and nitric oxide (NO), which not only exacerbate oxidative stress but also directly harm neurons and trigger neuronal death (83). Neuroinflammation further disrupts normal neuronal signaling, impairing synaptic function, and aggravating the progression of neurodegenerative diseases.

## 3 Nutrition and neurodegenerative diseases

### 3.1 AD

AD and metabolic dysregulation have emerged as a prominent focus in neurodegenerative research. Empirical evidence indicates that insulin resistance and mitochondrial impairment are critical pathogenic factors contributing to cognitive deterioration in AD. Insulin resistance not only disrupts cerebral metabolic homeostasis but also impairs synaptic plasticity, thereby facilitating neurodegeneration (84). Furthermore, mitochondrial dysfunction results in excessive reactive oxygen species (ROS) generation, intensifying neuronal injury (85).

Insulin resistance significantly mediates the link between AD and type 2 diabetes mellitus (T2DM). Epidemiological data reveal that over 80% of AD patients concurrently exhibit T2DM or dysglycemia, implying overlapping pathogenic pathways [2]. Insulin resistance exacerbates neuroinflammation, promotes *β*-amyloid aggregation, and induces abnormal tau phosphorylation, thereby accelerating AD progression (86, 87).

Additionally, the relationship between mitochondrial dysfunction, metabolic syndrome (MetS), and neurodegenerative disorders has garnered considerable scientific attention. Research suggests that MetS and diabetes share common mechanistic pathways in the pathogenesis of AD and related neurodegenerative conditions, with mitochondrial dynamics serving as a pivotal factor (85). Mitochondrial DNA mutations, environmental toxin exposure, and excessive caloric intake can precipitate mitochondrial impairment, subsequently leading to insulin resistance and elevated ROS production (85).

The Mediterranean diet, characterized by an abundance of vegetables, fruits, whole grains, nuts, olive oil, and fish, has been associated with a decreased risk of developing AD (88). This dietary pattern is rich in antioxidants, including vitamins C and E, as well as omega-3 polyunsaturated fatty acids, which possess anti-inflammatory and antioxidant properties that may mitigate neuronal damage linked to oxidative stress and neuroinflammation in AD (89). Additionally, the Mediterranean diet is high in fiber, which supports the health of the gut microbiome; the interplay between the gut microbiome and brain health is increasingly recognized and may influence AD progression by modulating inflammatory and metabolic pathways (89). Omega-3 polyunsaturated fatty acids, particularly DHA, hold significant promise in the prevention and management of AD (90). DHA is a crucial component of neuronal membranes, and its deficiency is associated with heightened neuroinflammation and neurodegenerative processes (91). Supplementation with DHA has been shown to enhance the stability of neuronal membranes, promote neuroprotection, and potentially improve cognitive function (92).

### 3.2 PD

Parkinson’s disease, as a complex neurodegenerative disorder, involves a pathogenic mechanism characterized by multifactorial processes, including oxidative stress, mitochondrial dysfunction, and lipid peroxidation. Recent research has increasingly elucidated the link between nutritional factors and neurodegeneration, particularly highlighting the impact of impaired oxidative phosphorylation and lipid peroxidation in PD pathogenesis.

Primarily, metabolic dysregulation in PD is closely associated with disturbances in glucose and lipid homeostasis. Evidence indicates that mitochondrial impairment may be connected to disruptions in lipid and glucose metabolism, as well as insulin resistance, collectively contributing to abnormal *α*-synuclein aggregation and degeneration of substantia nigra dopaminergic neurons (93). Furthermore, oxidative stress is a pivotal factor in neurodegeneration, with mitochondrial damage serving as a primary contributor to increased reactive oxygen species production and disease progression (94). Consequently, targeting these metabolic pathways presents promising therapeutic avenues for PD intervention.

Secondly, dysregulation of lipid metabolism significantly influences PD pathophysiology. Lipid peroxidation is a prevalent pathological hallmark in neurodegenerative conditions. Strategies aimed at inhibiting neuronal lipid oxidation may decelerate disease progression and mitigate symptom severity (95). Dietary antioxidants, such as polyphenols and vitamin-based antioxidants, may alleviate PD pathology by restoring redox homeostasis at the cellular level (96). Therefore, nutritional modulation and antioxidant supplementation could serve as effective strategies to delay PD progression.

Finally, nutritional interventions hold potential therapeutic value in PD management. Emerging studies demonstrate that nanocarrier systems, such as organic–inorganic hybrid mesoporous silica nanoparticles functionalized with bioactive compounds like curcumin and lactoferrin, can enhance neuroprotection and improve therapeutic efficacy through targeted delivery (97). Additionally, lipid-based nutrients play crucial roles in neuronal survival and neuroprotection in PD and AD, with prophylactic lipid supplementation potentially slowing disease advancement (98).

### 3.3 HD

HD is a hereditary neurodegenerative condition resulting from mutations in the gene responsible for encoding the Huntington protein, predominantly impacting the striatum region of the brain (99). This disorder leads to impairments in motor coordination, cognitive deterioration, and emotional disturbances (99). A significant factor in the pathogenesis of HD is the disruption of nutrient metabolism, particularly the abnormalities in energy metabolism, which not only compromise neuronal function but also correlate with the rate and severity of disease progression (100). Nutritional interventions, especially those aimed at modulating energy metabolism and oxidative stress, exhibit promising therapeutic potential for HD (101).

In HD patients, abnormal energy metabolism is characterized by diminished glucose metabolic efficiency, mitochondrial dysfunction, and impaired fatty acid oxidation (101, 102). These metabolic anomalies result in inadequate energy supply to neurons, further aggravating cellular damage. Mitochondria, which serve as the hub of energy metabolism, experience severe impairment in HD, evidenced by reduced oxidative phosphorylation efficiency and heightened reactive oxygen species (ROS) production, which damages mitochondrial DNA, instigates oxidative stress, and compromises neuronal structure and function (103, 104). Additionally, weight loss is frequently observed in HD patients, potentially linked to energy metabolism disorders and nutrient malabsorption (105).

### 3.4 Others

Amyotrophic Lateral Sclerosis (ALS) is characterized by the degeneration of motor neurons, leading to muscle weakness and atrophy (106). In individuals with ALS, there is a notable disruption in energy metabolism, evidenced by heightened energy expenditure coupled with diminished energy intake, which may contribute to muscle wasting and the elevated demands of neuronal function (107). Disorders in nutrient metabolism, particularly those affecting protein and amino acid pathways, play a crucial role in the progression of ALS (108). Implementing a high-protein diet and administering specific amino acid supplements, such as leucine, have demonstrated enhancements in muscle functionality and overall quality of life for ALS patients. Microbial therapy targeting the gut-brain axis (GBA) has also become an emerging personalized treatment approach (109). Furthermore, the incorporation of antioxidants and omega-3 fatty acids has been shown to confer neuroprotective benefits by mitigating oxidative stress and inflammation in this population (109).

Frontotemporal Dementia (FTD) encompasses a spectrum of disorders that primarily impact the frontal and temporal lobes of the brain, significantly influencing behavior, language, and cognitive abilities (110). In FTD patients, metabolic irregularities are evident through altered glucose metabolism, particularly within the frontotemporal regions (111). This metabolic dysfunction is linked to inadequate energy supply to neurons, thereby impairing neuronal activity and synaptic plasticity.

Furthermore, conditions such as Depression and Multiple Sclerosis (MS) are prevalent (112, 113). We have compiled a summary of nutrient metabolism irregularities and nutritional intervention strategies across various neurodegenerative disorders, as presented in Table 1.

**Table 1:** Nutrient metabolism abnormalities and nutritional intervention strategies in different neurodegenerative diseases.

Disease name	Nutrient metabolism abnormalities	Nutritional intervention strategies
Alzheimer’s disease	Lipid metabolism abnormalities: Associated with reduced intake of *ω*-3 fatty acids. Reduced antioxidant levels: Lower levels of antioxidants such as vitamin E.	Mediterranean and MIND diets: Rich in plant-based foods, olive oil, and fish, which may help reduce the risk of AD. ω-3 fatty acid supplementation: May help improve cognitive function. Antioxidant supplementation: Vitamins E and C may help reduce oxidative stress.
Parkinson’s disease	Oxidative stress and mitochondrial dysfunction: Associated with the damage of dopaminergic neurons.	Antioxidants: Vitamins C and E may help reduce oxidative stress. Foods rich in fiber and antioxidants: Fruits, vegetables, and whole grains.
Depression	Vitamin B deficiency: Particularly vitamins B6, B12, and folate.	Vitamin B supplementation: Particularly B6, B12, and folate, which may help improve symptoms. ω-3 fatty acid supplementation: May help alleviate depressive symptoms.
Multiple sclerosis	Lipid metabolism abnormalities: Related to an imbalance in the ratio of ω-3 to ω-6 fatty acids.	ω-3 fatty acid supplementation: May help regulate immune response and reduce inflammation. Low saturated fat diet: May help slow disease progression.
Amyotrophic lateral sclerosis	Reduced antioxidant levels: Associated with increased oxidative stress.	Antioxidant supplementation: Vitamins E and Coenzyme Q10 may help reduce oxidative stress. High-calorie, high-protein diet: Supports muscle function.
Huntington’s disease	Energy metabolism abnormalities: Related to mitochondrial dysfunction.	Ketogenic diet: May help provide an alternative energy source and improve energy metabolism. Coenzyme Q10 supplementation: May help improve mitochondrial function.
Frontotemporal dementia	Malnutrition and weight loss: Common in the progression of the disease.	Nutritional support: Providing high-calorie, high-protein diets to maintain weight and nutritional status. Probiotics and prebiotics: May help improve gut health and cognitive function.

## 4 Cell metabolic reprogramming and neurodegenerative diseases

### 4.1 Concept and mechanism of metabolic reprogramming

Metabolic reprogramming refers to the stimulus-induced rewiring of intracellular metabolic networks. Unlike tumor cells—where reprogramming supports biomass accumulation and uncontrolled proliferation—neurons prioritize survival and bioenergetic stability in response to chronic stress. In neurons, this process manifests as three core events: (i) a glycolytic shift toward aerobic glycolysis, (ii) impaired mitochondrial biogenesis and oxidative phosphorylation, and (iii) loss of redox homeostasis with sustained reactive oxygen species (ROS) generation. Collectively, these changes constitute an adaptive yet maladaptive response to persistent energy stress—initially protective, but ultimately driving oxidative damage and apoptotic signaling (18, 114–117).

However, neuronal metabolic reprogramming exhibits distinct characteristics (9). In contrast to tumor and immune cells, neurons are highly specialized and possess limited proliferative capacity, resulting in significant differences in the objectives and mechanisms underlying their metabolic reprogramming. In neurodegenerative diseases, this reprogramming is primarily characterized by aberrant energy metabolism, mitochondrial dysfunction, and alterations in nutrient utilization and demand, all of which directly impact neuronal viability and functionality (118).

In AD, a marked reduction in cerebral glucose metabolism serves as a critical indicator of metabolic reprogramming. This phenomenon not only signifies a diminished energy supply to the brain but is also intricately linked to neuronal injury and apoptosis (119). The decline in oxidative phosphorylation efficiency, attributed to mitochondrial dysfunction, is a pivotal contributor to the aberrant energy metabolism observed in neurons (120). Furthermore, insulin resistance plays a crucial role in AD, impairing glucose uptake and utilization by neurons, thereby exacerbating metabolic disturbances (121).

In PD, mitochondrial dysfunction and oxidative stress are central to the metabolic reprogramming process, resulting in the accumulation of reactive oxygen species (ROS) that further compromise neuronal integrity (122). In HD, abnormalities in energy metabolism are evident through diminished glucose metabolism efficiency, mitochondrial dysfunction, and impaired fatty acid oxidation, culminating in inadequate energy supply to neurons and exacerbating cellular damage.

The distinctiveness of neuronal metabolic reprogramming is also underscored by its intricate relationship with the pathological mechanisms of neurodegenerative diseases. For instance, in AD, the accumulation of *β*-amyloid plaques and neurofibrillary tangles not only disrupts neuronal structure and function but also interferes with normal metabolic pathways, instigating metabolic reprogramming (123). Similarly, in PD, the aggregation of *α*-synuclein adversely affects mitochondrial function, triggering metabolic reprogramming that intensifies oxidative stress and neuronal damage (124).

**Figure 2:**
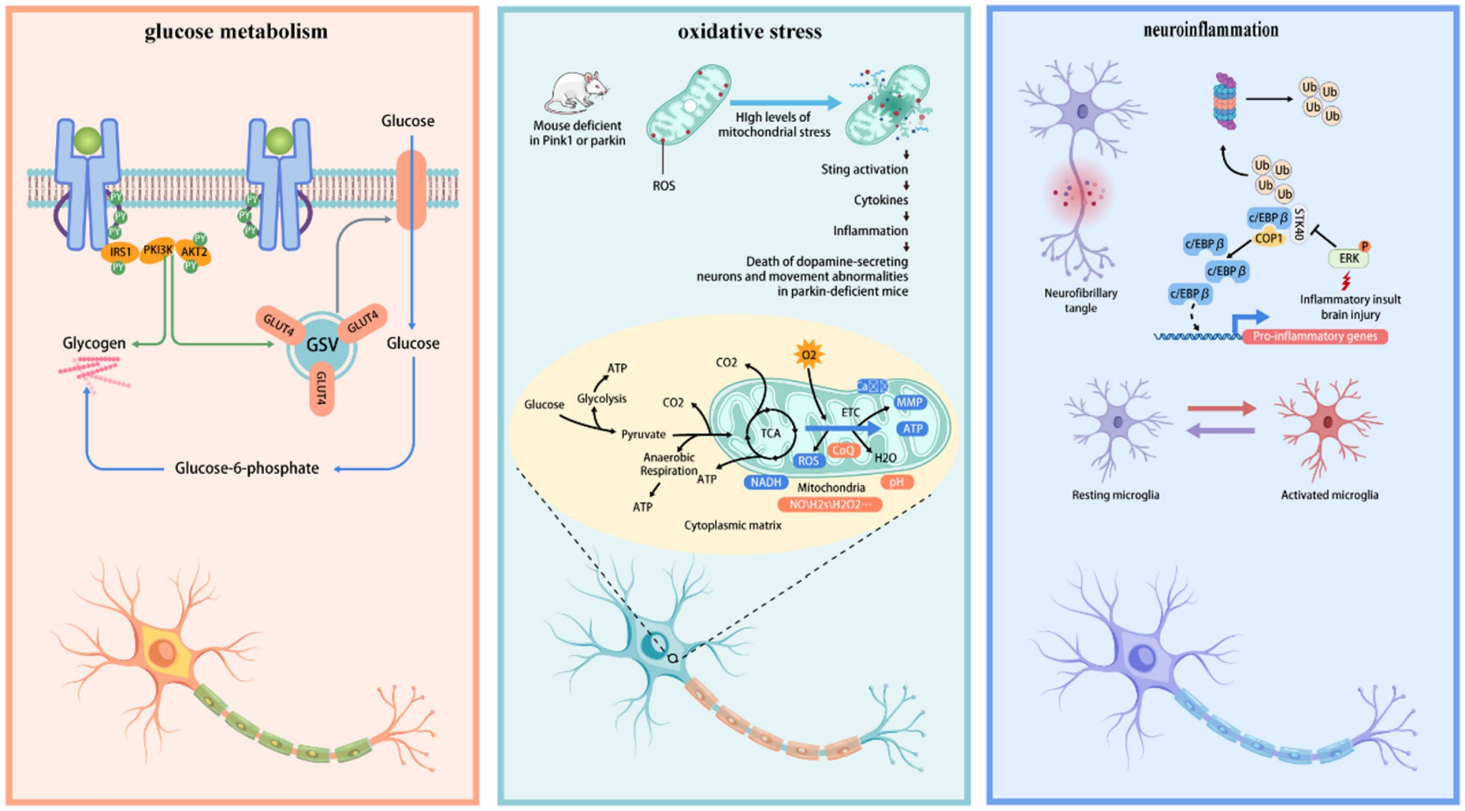
The mechanism of metabolic reprogramming in neurodegenerative diseases. Glucose enters neurons through the GLUT4 transporter, is converted into glucose-6-phosphate and pyruvic acid through glycolysis, provides initial energy, and can be converted into glycogen for storage. High levels of mitochondrial stress led to the production of reactive oxygen species (ROS), activate the STING pathway, trigger an inflammatory response, and cause the death and abnormal movement of dopamine-secreting neurons, especially evident in Parkin-deficient mice. Microglia are inactive at rest, but inflammatory stimulation or brain injury can activate them, promoting the expression of inflammatory genes through the c/EBPβ pathway and leading to the formation of neurofibrillary tangles.

Investigating the mechanisms of neuronal metabolic reprogramming is crucial for comprehending the fundamental nature of neurodegenerative diseases and formulating therapeutic interventions. The mechanisms involved encompass, but are not limited to, mitochondrial biogenesis and dysfunction, altered oxidative phosphorylation efficiency, modulation of glycolysis and tricarboxylic acid cycles, irregular fatty acid oxidation, and variations in nutrient utilization and demand by neurons. A thorough examination of these mechanisms will elucidate the specific contributions of metabolic reprogramming to neurodegenerative diseases, offering novel insights for diagnosis, prevention, and treatment strategies. We encapsulate the function of metabolic reprogramming in neurodegenerative disorders in Figure 2.

### 4.2 Regulation of metabolic reprogramming

The potential of metabolic reprogramming as a regulatory and therapeutic target offers extensive research opportunities within the domain of neurodegenerative diseases (125). Both nutritional and pharmacological interventions can significantly influence neuronal survival and functionality by modulating critical metabolic pathways, as well as regulating neuroinflammation and oxidative stress, thereby potentially decelerating or reversing neurodegenerative processes (18, 126).

Nutritional interventions, particularly those involving diets abundant in antioxidants, omega-3 polyunsaturated fatty acids, and specific amino acids, exert a profound regulatory influence on metabolic reprogramming (127, 128). The Mediterranean diet, characterized by its rich variety of vegetables, fruits, whole grains, nuts, olive oil, and fish, has demonstrated efficacy in enhancing energy metabolism, mitigating oxidative stress and inflammation, and providing neuroprotective benefits (129). Antioxidants found in the Mediterranean diet, such as vitamin C, vitamin E, and selenium, play a crucial role in neutralizing free radicals, alleviating oxidative stress, and safeguarding neuronal integrity (130). Omega-3 polyunsaturated fatty acids, particularly DHA and EPA, not only diminish the synthesis of inflammatory mediators but also enhance mitochondrial functionality, improve neuronal membrane fluidity, facilitate signaling processes, and hold potential therapeutic value for neurodegenerative conditions (131). Tailored nutritional interventions, customized according to individual metabolic profiles and disease conditions, can more effectively modulate neuronal metabolism, decrease oxidative stress and inflammation, and foster neuroprotection by optimizing dietary components, such as increasing the intake of antioxidants and fatty acids. For instance, in patients with neurodegenerative diseases characterized by mitochondrial dysfunction, the supplementation of antioxidants and omega-3 fatty acids may yield superior neuroprotective outcomes (132).

Pharmacological interventions, particularly those aimed at specific metabolic pathways, also exhibit promising regulatory effects on metabolic reprogramming. For example, insulin sensitizers like thiazolidinediones confer neuroprotective benefits in neurodegenerative diseases by enhancing insulin sensitivity and glucose metabolism (133). These agents facilitate glucose uptake and utilization, thereby improving the energy supply to neurons and potentially slowing disease progression. Antioxidants and anti-inflammatory medications, such as N-Acetylcysteine amide and non-steroidal anti-inflammatory drugs, provide protective effects against neurodegenerative diseases by diminishing oxidative stress and curtailing inflammation (134, 135). These pharmacological agents can lower the production of reactive oxygen species (ROS), inhibit the release of inflammatory mediators, and mitigate neuronal damage.

The pivotal function of metabolic reprogramming in neurodegenerative pathologies underscores its significance as a therapeutic target. Notably, modulation of the AMPK/PGC-1α signaling axis has been extensively investigated. Evidence indicates that Dihuang Yinzi enhances mitochondrial biogenesis via this pathway, thereby ameliorating cognitive deficits and mitochondrial ultrastructural damage in transgenic Alzheimer’s disease models (136). Furthermore, Sestrin2 overexpression mitigates osteoarthritic pain and suppresses neuroinflammatory responses through activation of the AMPK/PGC-1α pathway (137). These findings elucidate the therapeutic potential of targeting the AMPK/PGC-1α signaling cascade in neurodegenerative and neuroinflammatory disorders.

## 5 Nutritional interventions and therapeutic strategies

### 5.1 Emerging interventions integrate dietary modification, probiotics, and pharmacologic agents

**Figure 3:**
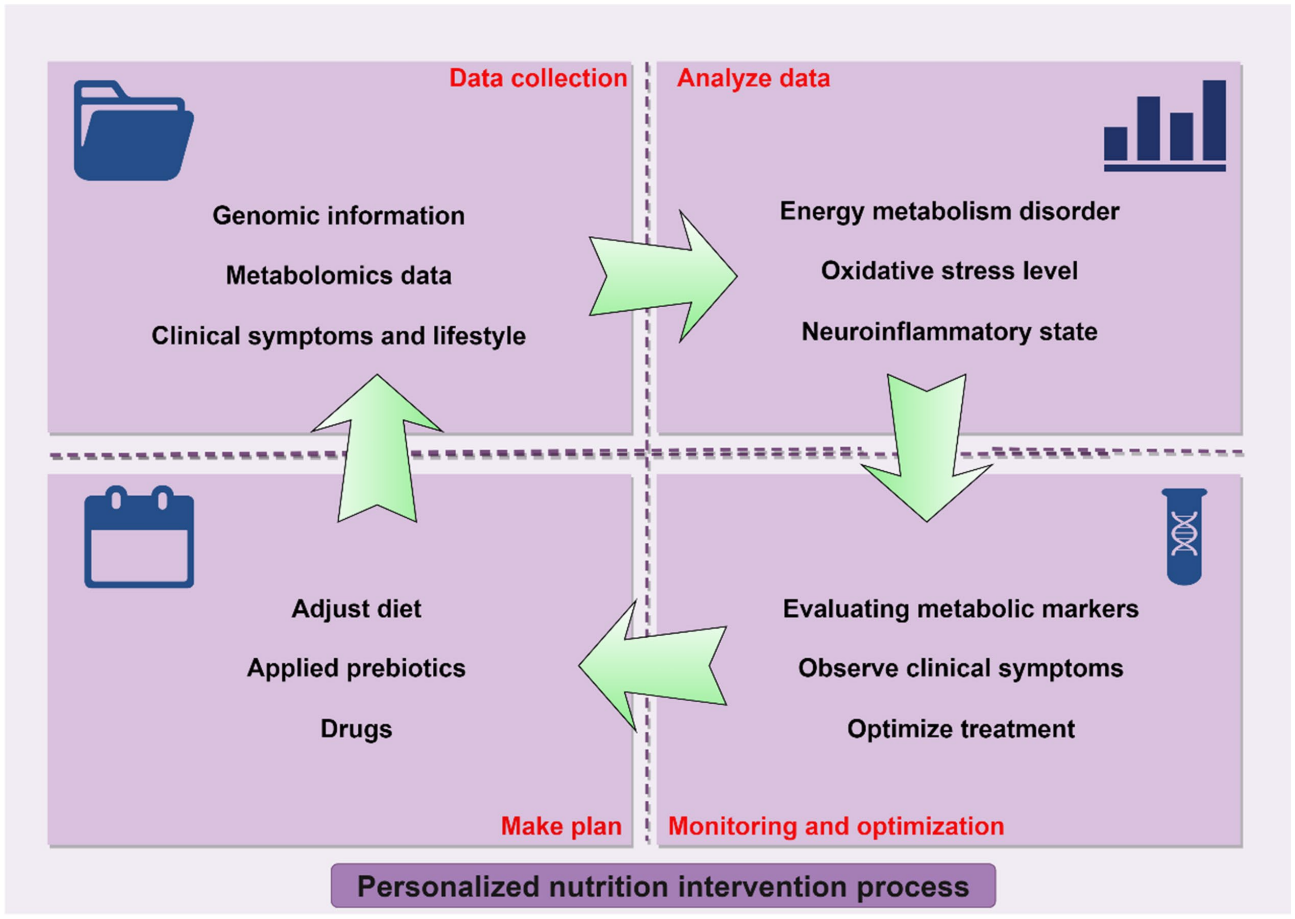
Personalized nutrition intervention strategies.

Currently, there is an increasing focus on the role of nutritional interventions in the management of neurodegenerative diseases, highlighting their significant potential (138). Nutrients, particularly antioxidants, omega-3 polyunsaturated fatty acids, vitamins, minerals, and specific amino acids, exhibit neuroprotective properties by enhancing energy metabolism and mitigating oxidative stress and inflammation. The Mediterranean diet, abundant in these advantageous components, has demonstrated improvements in cognitive function and a reduction in disease symptoms among individuals with neurodegenerative conditions (139). Tailored nutritional intervention strategies, which adjust dietary plans to align with the unique metabolic requirements of patients, further amplify the efficacy of these interventions. Nonetheless, nutritional interventions encounter several challenges and limitations in the context of neurodegenerative disease treatment. Therefore, we summarized the advantages and challenges of nutritional therapy in the treatment of neurodegenerative diseases in Table 2, and we present customized nutritional intervention protocols depicted in Figure 3.

**Table 2:** Advantages and challenges of nutritional intervention in the treatment of neurodegenerative diseases.

Advantages	Limitations
1. *High safety*: Nutritional interventions typically use natural foods or supplements, resulting in fewer side effects and being suitable for long-term use.	1. *Lack of standardized protocols*: There is currently a lack of unified guidelines for nutritional interventions, leading to inconsistent results across different studies and making it difficult to establish standardized treatment protocols.
2. *Improves overall health*: By enhancing nutritional status, it boosts immune function and quality of life.	2. *Patient compliance*: Long-term adherence to nutritional interventions may require changes in dietary habits, which some patients may find difficult to maintain.
3. *Slows disease progression*: Certain nutrients (such as ω-3 fatty acids and antioxidants) may help reduce neuroinflammation and oxidative stress, thereby slowing disease progression.	3. *Nutrient absorption issues*: Some patients with neurodegenerative diseases may experience problems with nutrient absorption, affecting the effectiveness of the intervention.
4. *Personalized treatment*: Nutritional interventions can be tailored to the patient’s metabolic characteristics and nutritional needs, improving treatment outcomes.	4. *Complex mechanisms of action*: The mechanisms of action of nutrients are complex and may involve multiple biological pathways, making it difficult to pinpoint their exact mechanisms and optimal dosages.
5. *Multi-target action*: Nutritional interventions can affect disease progression through multiple pathways, such as antioxidant, anti-inflammatory, and mitochondrial function improvement.	5. *Insufficient research evidence*: Although some studies support the effectiveness of nutritional interventions, the overall strength of evidence is still insufficient, necessitating more large-scale, long-term studies.
6. *Synergistic with other treatments*: Nutritional interventions can work synergistically with other treatments (such as medication and physical therapy) to enhance overall treatment efficacy.	6. *Individual differences*: Patients may respond differently to nutritional interventions, requiring adjustments based on individual needs.
7. *Enhances quality of life*: By improving nutritional status, patients’ quality of life can be significantly enhanced.	7. *Disease complexity*: Neurodegenerative diseases often involve multiple pathophysiological mechanisms, and a single nutritional intervention may not be sufficient to address all aspects of the disease.
	8. *Limited efficacy*: Nutritional interventions cannot replace traditional medication and primarily serve as an adjunctive therapy.
	9. *Uncertain long-term effects*: The long-term efficacy and safety of nutritional interventions require further research.
	10. *Economic costs*: Some nutritional supplements and special diets can be costly, making them unaffordable for some patients.
	11. *Cultural and dietary habits*: Cultural and dietary habits in different regions may influence the implementation and effectiveness of nutritional interventions.

In the context of neurodegenerative diseases, the utilization of probiotics seeks to restore balance to the gut microbiome, thereby influencing central nervous system health via the microbial-gut-brain axis (140). Probiotics, including Lactobacillus and Bifidobacterium, exhibit neuroprotective properties by inhibiting pathogenic growth, enhancing intestinal barrier integrity, modulating immune responses, and alleviating oxidative stress (141). Furthermore, they play a crucial role in the synthesis and metabolism of neurotransmitters like serotonin and dopamine, which are vital for mood regulation, memory, and motor function (142). Postbiotic metabolites, derived from dead probiotics, continue to offer health benefits. In contrast to live probiotics, postbiotics demonstrate enhanced stability and safety, particularly following antibiotic therapy or in individuals with compromised immune systems. These metabolites, such as short-chain fatty acids (SCFAs), positively influence gut and brain health (143).

**Figure 4:**
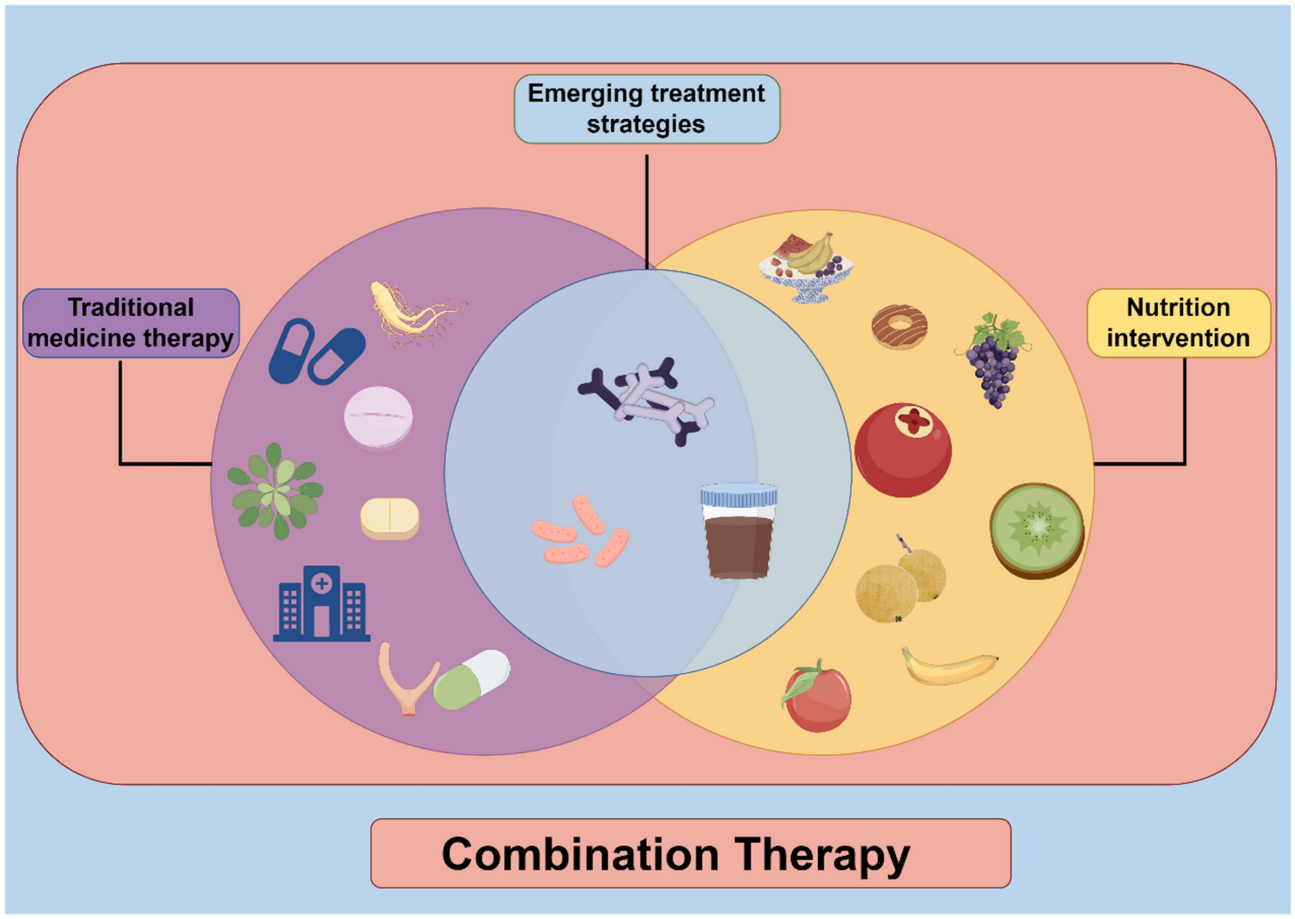
Therapeutic strategies for neurodegenerative diseases combined with nutritional intervention.

The strategy of combining nutrients and pharmaceuticals aims to synergistically amplify therapeutic outcomes, mitigate drug side effects, and enhance patient quality of life. We present a synthesis of nutritional interventions alongside treatment strategies for neurodegenerative diseases in Figure 4. Additionally, we compiled a summary of the emerging nutritional intervention strategies along with their mechanisms of action in Table 3. Advancements in targeted delivery systems for neurodegenerative disorders have garnered considerable research focus, particularly in the utilization of nanotechnology for precision therapeutics. The development of diverse nanocarriers—including polymeric nanoparticles, liposomes, inorganic nanomaterials, and biomimetic vectors—has enabled brain-specific drug delivery and localized release via stimulus-responsive nanocarrier platforms (144). Furthermore, mitochondrial-targeted nanotherapeutics demonstrate substantial promise in neurodegeneration management; employing carriers such as liposomes, DQAsomes, and polymeric nanoparticles enhances mitochondrial drug delivery, thereby increasing bioavailability and therapeutic efficacy (145). Recent progress has also been achieved in overcoming the blood–brain barrier (BBB). Techniques such as ligand-functionalized nanoparticles, bispecific antibody shuttles, focused ultrasound-mediated BBB modulation, intranasal administration, exosomes, and mRNA lipid nanoparticles facilitate effective central nervous system drug delivery (146). The naso-brain delivery pathway, an innovative approach, enables direct transport of therapeutics to the brain via olfactory or trigeminal nerve pathways, bypassing the BBB, which enhances pharmacological efficacy and minimizes systemic adverse effects. This method supports the integration of nanotheranostic systems for more precise diagnosis and personalized treatment of neurodegenerative diseases (147).

**Table 3:** Emerging nutrition intervention strategies and their mechanisms of action.

Emerging nutritional intervention strategies	Mechanisms of action
1. Ketogenic diet	*Energy metabolism regulation*: By reducing carbohydrate intake, the body is forced to use fat as the primary energy source, producing ketones, which enhances mitochondrial function and energy metabolism. *Neuroprotective effects*: Ketones possess antioxidant and anti-inflammatory properties, which may help protect neurons from damage.
2. Omega-3 fatty acids supplementation	*Anti-inflammatory effects*: Omega-3 fatty acids (such as EPA and DHA) reduce neuroinflammation by inhibiting the production of pro-inflammatory cytokines. *Cell membrane stability*: They improve the fluidity and function of neuronal cell membranes, enhancing neural signal transmission.
3. Intermittent fasting	*Autophagy activation*: Through periodic fasting, autophagy is induced to clear damaged proteins and organelles, improving cellular health. *Metabolic regulation*: It enhances insulin sensitivity and reduces oxidative stress and inflammation levels.
4. Probiotics and prebiotics	*Gut-brain axis modulation*: By improving the balance of the gut microbiota, it affects the production of neurotransmitters (such as serotonin), regulating mood and behavior. *Immune regulation*: It enhances immune function and reduces inflammatory responses.
5. Curcumin	*Antioxidant and anti-inflammatory effects*: Curcumin has strong antioxidant and anti-inflammatory properties, inhibiting neuroinflammation and oxidative stress. *Protein aggregation inhibition*: It inhibits the aggregation of β-amyloid and tau proteins, reducing neurotoxicity.
6. Coenzyme Q10	*Mitochondrial function support*: Coenzyme Q10 is a crucial component of the mitochondrial electron transport chain, supporting ATP production and reducing oxidative stress. *Antioxidant effects*: It scavenges free radicals, protecting cells from oxidative damage.
7. Resveratrol	*SIRT1 activation*: By activating the longevity gene SIRT1, it regulates cellular metabolism and stress responses, delaying cellular aging. Anti-inflammatory and *Antioxidant effects*: It inhibits inflammatory factors and oxidative stress, improving cellular health.
8. Vitamin D	*Immune regulation*: It regulates the function of immune cells, reducing autoimmune responses. *Neuroprotective effects*: It promotes the production of nerve growth factors, protecting neurons.
9. Alpha-lipoic acid	*Antioxidant effects*: As a potent antioxidant, it scavenges free radicals, reducing oxidative stress. *Metabolic regulation*: It improves glucose metabolism and insulin sensitivity.
10. Nicotinamide riboside	*NAD+ level enhancement*: It increases intracellular NAD + levels, activating energy metabolism and cellular repair mechanisms. *Anti-aging effects*: By regulating cellular metabolism, it delays the aging process.

### 5.2 Clinical trials and future directions

To validate the efficacy of nutritional interventions for neurodegenerative diseases, it is imperative that clinical trials are meticulously structured to guarantee the precision and dependability of the findings. A well-defined research objective is essential, specifying the intended outcomes of the nutritional intervention, such as enhancing cognitive function, alleviating disease symptoms, or slowing disease progression. Subsequently, an appropriate control group must be established to evaluate the true impact of the intervention. This control group may consist of patients receiving standard treatment or those undergoing a simulated nutritional intervention (e.g., a placebo) to eliminate the effects of extraneous variables.

Furthermore, the design of clinical trials must consider the intervention’s duration, dosage, and the specific nutrients involved. The timing of the intervention should be in-formed by prior research or theoretical frameworks to identify the optimal moment for nutritional intervention. Dosage selection should rely on safety and efficacy data from earlier studies to ensure both safety and effectiveness. Additionally, the choice of nutrients should be guided by their mechanisms of action in neurodegenerative conditions and findings from previous investigations.

Recent evidence has further substantiated the potential of dietary interventions in modulating neurodegenerative pathways. For instance, Mafe and Büsselberg demonstrated that adherence to a Mediterranean diet (MedDiet) significantly altered gut microbiota composition and associated metabolite signatures in patients with mild cognitive impairment (MCI), suggesting a plausible mechanistic link between diet-induced microbial changes and attenuated Alzheimer’s disease (AD) progression (148). Their findings align with our proposed framework that MedDiet-mediated metabolic reprogramming may exert neuroprotective effects via gut–brain axis modulation.

Similarly, Orywal et al. provided comprehensive preclinical and clinical data supporting the role of B-group vitamins, particularly B6, B12, and folate, in mitigating oxidative stress and homocysteine-mediated neurotoxicity across AD and Parkinson’s disease (PD) cohorts (149). These results reinforce our advocacy for personalized nutritional strategies that integrate micronutrient status assessment, especially in early-stage neurodegeneration.

Collectively, these studies underscore the necessity of designing multi-arm, diet-centric clinical trials that not only assess cognitive endpoints but also incorporate multi-omics readouts (microbiome, metabolome, epigenome) to capture the full therapeutic trajectory of nutritional interventions.

To enhance the scientific rigor and applicability of the study, a multicenter, randomized, double-blind, placebo-controlled design is recommended. A multicenter approach can augment sample size and the generalizability of results, while randomization minimizes selection bias. The double-blind methodology helps to mitigate subjective influences from both investigators and participants, and placebo controls are essential for assessing the genuine effects of the intervention. Moreover, incorporating biomarkers as objective measures of intervention outcomes, such as neuronal metabolites and oxidative stress markers, can provide more robust evidence to support the findings. To ensure adequate statistical power, future trials should pre-calculate sample size on the basis of clinically relevant effect sizes of cognitive or imaging endpoints, typically requiring 50–100 participants per arm for phase IIa studies. Core biomarker panels ought to include plasma Aβ42/40 ratio, p-tau-181, neurofilament light chain, and short-chain fatty acids quantified by targeted metabolomics, which have shown responsiveness to Mediterranean diet or omega-3 interventions and correlate with cognitive change.

## 6 Discussion

This review systematically elucidates the critical function of nutritional modulation and cellular metabolic reprogramming in neurodegenerative pathologies: dysregulation of glucose, lipid, and amino acid metabolism collectively precipitates energy deficits, oxidative stress, and neuroinflammatory responses, establishing a “metabolism-inflammation” axis. Interventions such as the Mediterranean diet, omega-3 polyunsaturated fatty acids, and B vitamin supplementation can markedly enhance cognitive performance and attenuate peripheral inflammatory biomarkers by restoring mitochondrial bioenergetics, bolstering antioxidant defenses, and modulating the gut-brain axis, thereby forming a closed-loop mechanistic framework for the “nutrition-metabolism-neuroprotection” triad.

Current evidence is limited by four primary deficiencies: ① substantial heterogeneity across studies due to variable dosing, intervention durations, and baseline nutritional statuses, complicating meta-analyses; ② small sample sizes, often fewer than 50 participants per group, lacking statistical power calculations and increasing the risk of Type II errors; ③ reliance on cognitive assessment scales such as MMSE and MoCA without concurrent biomarker evaluation (e.g., Aβ42/40 ratios, phosphorylated tau-181, neurofilament light chain, short-chain fatty acids); ④ paucity of long-term safety and adherence data, with research predominantly focused on Alzheimer’s disease, leaving mechanisms in other neurodegenerative disorders insufficiently characterized.

Future research should prioritize multicenter, randomized, double-blind, placebo-controlled trials with pre-specified sample size calculations based on clinically meaningful endpoints (e.g., CDR-SB ≥ 0.5 or hippocampal atrophy ≥3% annually), ensuring at least 50–100 participants per arm in phase IIA studies. Furthermore, developing a standardized core biomarker panel comprising plasma Aβ42/40 ratio, phosphorylated tau-181, neurofilament light chain (NfL), and targeted short-chain fatty acid metabolomics will function as dual endpoints for assessing therapeutic response and mechanistic validation. Integrating multi-omics data and artificial intelligence predictive modeling will facilitate personalized nutritional strategies, with extended follow-up periods of ≥24 months to assess disease-modifying potential. Ultimately, the goal is to translate the concept of “nutrition-driven metabolic reprogramming” from observational phenomena into a clinically applicable intervention paradigm for neurodegenerative disease management.

## 7 Conclusion

Neurodegenerative disorders are increasingly understood as complex, multifactorial pathologies driven by metabolic dysregulation, oxidative stress, and neuroinflammatory processes. This review emphasizes the critical influence of nutritional status and cellular metabolic reprogramming on the etiology and progression of diseases such as AD, PD, and HD. Growing evidence indicates the therapeutic potential of dietary interventions—particularly those enriched with antioxidants, omega-3 polyunsaturated fatty acids, and B vitamins—in modulating neurobiological metabolic pathways, improving mitochondrial bioenergetics, and reducing neuroinflammation. Despite encouraging preclinical and clinical results, the clinical integration of nutritional therapeutic strategies faces challenges due to variability in study methodologies, lack of standardized intervention protocols, and inadequate biomarker incorporation. Future investigations should focus on large-scale, multicenter randomized controlled trials utilizing multi-omics technologies and personalized nutrition approaches. Tailoring dietary regimens to individual metabolic phenotypes holds promise for developing safe, effective, and sustainable adjunctive treatments in neurodegenerative disease management. Ultimately, elucidating the connections between nutrition and brain energy metabolism is essential for advancing mechanistic understanding and for establishing precision nutrition as a cornerstone of neurodegeneration therapeutics.
